# {2,2′-[*o*-Phenyl­enebis(nitrilo­methanylyl­idene)]diphenolato-κ^4^ *O*,*N*,*N*′,*O*′}nickel(II) monohydrate

**DOI:** 10.1107/S1600536811039730

**Published:** 2011-10-05

**Authors:** Akbar Ghaemi, Kazem Fayyazi, Bahram Keyvani, Seik Weng Ng, Edward R. T. Tiekink

**Affiliations:** a Department of Chemistry, Saveh Branch, Islamic Azad University, Saveh, Iran; b Department of Chemistry, University of Malaya, 50603 Kuala Lumpur, Malaysia; c Chemistry Department, Faculty of Science, King Abdulaziz University, PO Box 80203 Jeddah, Saudi Arabia

## Abstract

The Ni^II^ atom in the title monohydrate, [Ni(C_20_H_14_N_2_O_2_)]·H_2_O, is coordinated within a *cis*-N_2_O_2_ square-planar donor set provided by the tetra­dentate Schiff base ligand. Overall, the mol­ecule has a curved shape with the dihedral angle formed between the planes of the outer benzene rings being 13.92 (18)°. The water mol­ecule was found to be disordered over two positions [ratio 0.80 (1):0.20 (1)] and the major component is linked to the complex *via* an O—H⋯O hydrogen bond.

## Related literature

For background to the catalytic potential of transition metal Schiff base complexes, see: Gupta & Sutar (2008) ▶. For the structure of the unsolvated form of the title complex. see: Radha *et al.* (1985 ▶); Wang *et al.* (2003 ▶). For our recent work in this area, see: Ghaemi *et al.* (2011 ▶).
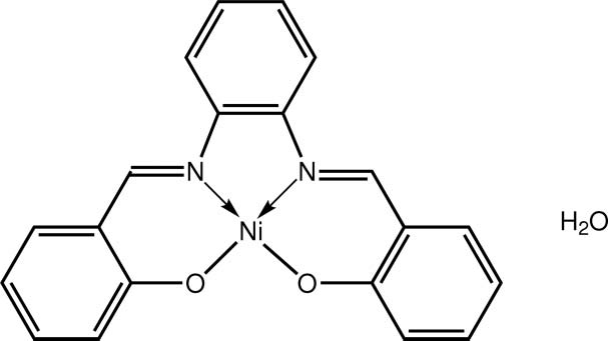


## Experimental

### 

#### Crystal data

[Ni(C_20_H_14_N_2_O_2_)]·H_2_O
*M* *_r_* = 391.06
Trigonal, 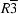

*a* = 31.5519 (13) Å
*c* = 9.0255 (6) Å
*V* = 7781.3 (6) Å^3^
*Z* = 18
Mo *K*α radiation
μ = 1.14 mm^−1^
*T* = 294 K
0.30 × 0.15 × 0.15 mm

#### Data collection

Agilent SuperNova Dual diffractometer with an Atlas detector
Absorption correction: multi-scan (*CrysAlis PRO*; Agilent, 2010 ▶) *T*_min_ = 0.732, *T*_max_ = 1.0
13452 measured reflections
3897 independent reflections
2850 reflections with *I* > 2σ(*I*)
*R*_int_ = 0.036

#### Refinement

*R*[*F* ^2^ > 2σ(*F* ^2^)] = 0.041
*wR*(*F* ^2^) = 0.120
*S* = 1.04
3897 reflections
251 parameters
3 restraints
H atoms treated by a mixture of independent and constrained refinement
Δρ_max_ = 0.52 e Å^−3^
Δρ_min_ = −0.43 e Å^−3^

### 

Data collection: *CrysAlis PRO* (Agilent, 2010 ▶); cell refinement: *CrysAlis PRO*; data reduction: *CrysAlis PRO*; program(s) used to solve structure: *SHELXS97* (Sheldrick, 2008 ▶); program(s) used to refine structure: *SHELXL97* (Sheldrick, 2008 ▶); molecular graphics: *ORTEP-3* (Farrugia, 1997 ▶) and *DIAMOND* (Brandenburg, 2006 ▶); software used to prepare material for publication: *publCIF* (Westrip, 2010 ▶).

## Supplementary Material

Crystal structure: contains datablock(s) global, I. DOI: 10.1107/S1600536811039730/hb6421sup1.cif

Structure factors: contains datablock(s) I. DOI: 10.1107/S1600536811039730/hb6421Isup2.hkl

Additional supplementary materials:  crystallographic information; 3D view; checkCIF report

## Figures and Tables

**Table 1:** Selected bond lengths (Å)

Ni—O1	1.8865 (18)
Ni—O2	1.886 (2)
Ni—N1	1.930 (2)
Ni—N2	1.935 (2)

**Table 2:** Hydrogen-bond geometry (Å, °)

*D*—H⋯*A*	*D*—H	H⋯*A*	*D*⋯*A*	*D*—H⋯*A*
O1w—H1⋯O1	0.83 (1)	2.06 (2)	2.842 (4)	158 (5)

**Fig. 1.**
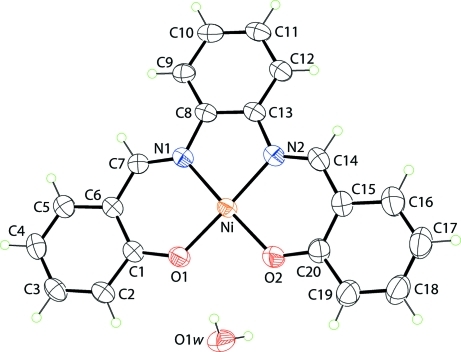
The molecular structure of (I) showing displacement ellipsoids at the 35% probability level. Only the major component of the disordered water molecule is illustrated.

## supplementary crystallographic information

### Comment

Schiff base complexes of transition metal ions are efficient catalysts both in homo- and hetero-geneous reactions, and the activity of these complexes varies with the type of ligand, coordination sites and metal ions (Gupta & Sutar, 2008). In continuation of work in this area (Ghaemi *et al.*, 2011), the title complex, (I), was isolated as a monohydrate and characterized crystallographically. An unsolvated form has been characterized previously (Radha *et al.*, 1985; Wang *et al.*, 2003).

The Ni^II^ atom in the complex exists within a *cis*-N_2_O_2_ donor set defined by the tetradentate Schiff base ligand, Fig. 1 and Table 1. The respective pairs of Ni—O and Ni—N bond distances are equal within experimental error. The greatest deviation from the ideal square planar angles is seen in the N1—Ni—N2 chelate angle of 83.98 (9)°. Some minor buckling is found in the N_2_O_2_ donor set with the r.m.s. deviation being 0.0548 Å. The maximum deviations from the least-squares plane are 0.0550 (10) and -0.0552 (10) Å for the N1 and N2 atoms, respectively, and the Ni atom lies 0.0002 (11) Å out of the least-squares plane. Each of the chelate rings is essentially planar. Thus, the r.m.s. deviation for the five-membered ring is 0.046 Å, and the equivalent values for the O1- and O2-containing six-membered chelate rings are 0.013 and 0.097 Å, respectively. The dihedral angle formed between the outer benzene rings is 13.92 (18)°, indicating that overall the molecule has a slightly curved shape.

The water molecule of solvation is associated with the complex molecule, forming a hydrogen bond with the O1 atom. Disorder in the position of the water molecule precludes a detailed analysis of the supramolecular structure.

### Experimental

*N*,*N*'-Bis(2-hydroxybenzylidene)-*o*-phenylenediamine was prepared by the following procedure. To a stirred ethanolic solution (30 ml) of *o*-phenylenediamine (0.108 g, 1 mmol), 2-hydroxybenzaldehyde (0.244 g, 2 mmol) was added. The bright-yellow solution was stirred and heated to reflux for 1 h. A yellow precipitate was obtained that was filtered off, washed with diethyl ether; yield: 75%. The title complex was obtained by the following procedure. The Schiff base ligand (0.316 g, 1 mmol) was dissolved in 20 ml e thanol. A solution of nickel(II) acetate (0.248 g, 1 mmol) in ethanol was added to the solution of ligand and the reaction mixture was refluxed for 1 h. The product washed with ethanol and air dried; yield: 85%. Dark brown blocks of the title complex were obtained from its 5:1 acetone and methanol mixture (*v*/*v*) by slow evaporation of the solvents at room temperature over several days.

### Refinement

The C-bound H-atoms were placed in calculated positions (C—H 0.93 Å) and were included in the refinement in the riding model approximation, with *U*_iso_(H) set to 1.2*U*_equiv_(C). The water molecule is disordered over two positions in a 0.80 (1):0.20 (1) ratio (from refinement). The H atoms were found for the major component only. These were very tightly restrained with O–H = 0.84±0.01 Å and H···H = 1.37 + 0.01 Å; *U*_iso_(H) was set to 1.5*U*_equiv_(O). The major component forms a hydrogen bond (through the H1 atom), but the H2 atom occupies a site close to that occupied by the minor component.

### Crystal data

**Table:**

[Ni(C_20_H_14_N_2_O_2_)]·H_2_O	*D*_x_ = 1.502 Mg m^−^^3^
*M**_r_* = 391.06	Mo *K*α radiation, λ = 0.71073 Å
Trigonal, *R*3	Cell parameters from 4020 reflections
Hall symbol: -R 3	θ = 2.2–29.2°
*a* = 31.5519 (13) Å	µ = 1.14 mm^−^^1^
*c* = 9.0255 (6) Å	*T* = 294 K
*V* = 7781.3 (6) Å^3^	Block, dark-brown
*Z* = 18	0.30 × 0.15 × 0.15 mm
*F*(000) = 3636	

### Data collection

**Table:**

Agilent SuperNova Dual diffractometer with an Atlas detector	3897 independent reflections
Radiation source: SuperNova (Mo) X-ray Source	2850 reflections with *I* > 2σ(*I*)
Mirror	*R*_int_ = 0.036
Detector resolution: 10.4041 pixels mm^-1^	θ_max_ = 27.5°, θ_min_ = 2.4°
ω scans	*h* = −40→39
Absorption correction: multi-scan (*CrysAlis PRO*; Agilent, 2010)	*k* = −30→40
*T*_min_ = 0.732, *T*_max_ = 1.0	*l* = −11→11
13452 measured reflections	

### Refinement

**Table:**

Refinement on *F*^2^	Primary atom site location: structure-invariant direct methods
Least-squares matrix: full	Secondary atom site location: difference Fourier map
*R*[*F*^2^ > 2σ(*F*^2^)] = 0.041	Hydrogen site location: inferred from neighbouring sites
*wR*(*F*^2^) = 0.120	H atoms treated by a mixture of independent and constrained refinement
*S* = 1.04	*w* = 1/[σ^2^(*F*_o_^2^) + (0.0632*P*)^2^ + 2.9091*P*] where *P* = (*F*_o_^2^ + 2*F*_c_^2^)/3
3897 reflections	(Δ/σ)_max_ < 0.001
251 parameters	Δρ_max_ = 0.52 e Å^−^^3^
3 restraints	Δρ_min_ = −0.43 e Å^−^^3^

### Special details

**Table:**

Geometry. All e.s.d.'s (except the e.s.d. in the dihedral angle between two l.s. planes) are estimated using the full covariance matrix. The cell e.s.d.'s are taken into account individually in the estimation of e.s.d.'s in distances, angles and torsion angles; correlations between e.s.d.'s in cell parameters are only used when they are defined by crystal symmetry. An approximate (isotropic) treatment of cell e.s.d.'s is used for estimating e.s.d.'s involving l.s. planes.
Refinement. Refinement of *F*^2^ against ALL reflections. The weighted *R*-factor *wR* and goodness of fit *S* are based on *F*^2^, conventional *R*-factors *R* are based on *F*, with *F* set to zero for negative *F*^2^. The threshold expression of *F*^2^ > σ(*F*^2^) is used only for calculating *R*-factors(gt) *etc*. and is not relevant to the choice of reflections for refinement. *R*-factors based on *F*^2^ are statistically about twice as large as those based on *F*, and *R*- factors based on ALL data will be even larger.

### Fractional atomic coordinates and isotropic or equivalent isotropic displacement parameters (Å^2^)

**Table:**

	*x*	*y*	*z*	*U*_iso_*/*U*_eq_	Occ. (<1)
Ni	0.517165 (12)	0.069858 (12)	0.75915 (3)	0.04310 (14)	
O1	0.46763 (7)	0.05129 (7)	0.9031 (2)	0.0534 (5)	
O2	0.53199 (8)	0.13507 (8)	0.7881 (2)	0.0663 (6)	
O1W	0.49982 (18)	0.12723 (16)	1.1173 (4)	0.0839 (16)	0.799 (10)
O1W'	0.5396 (7)	0.1609 (5)	1.128 (2)	0.081 (6)	0.201 (10)
H1	0.4901 (17)	0.1108 (15)	1.040 (3)	0.122*	
H2	0.5295 (9)	0.149 (2)	1.109 (7)	0.122*	
N1	0.50665 (8)	0.00460 (8)	0.7325 (2)	0.0433 (5)	
N2	0.56480 (8)	0.08656 (8)	0.6025 (2)	0.0461 (5)	
C1	0.43849 (9)	0.00727 (10)	0.9530 (3)	0.0442 (6)	
C2	0.40283 (10)	0.00071 (12)	1.0577 (3)	0.0518 (7)	
H2A	0.4003	0.0276	1.0871	0.062*	
C3	0.37181 (10)	−0.04400 (12)	1.1177 (3)	0.0558 (7)	
H3	0.3485	−0.0471	1.1865	0.067*	
C4	0.37476 (11)	−0.08475 (12)	1.0770 (3)	0.0594 (8)	
H4	0.3539	−0.1151	1.1191	0.071*	
C5	0.40858 (10)	−0.07978 (12)	0.9743 (3)	0.0559 (7)	
H5	0.4104	−0.1072	0.9465	0.067*	
C6	0.44089 (10)	−0.03438 (10)	0.9090 (3)	0.0454 (6)	
C7	0.47473 (10)	−0.03337 (10)	0.8036 (3)	0.0457 (6)	
H7	0.4738	−0.0628	0.7842	0.055*	
C8	0.53948 (9)	0.00265 (10)	0.6283 (3)	0.0440 (6)	
C9	0.54204 (11)	−0.03870 (11)	0.5953 (3)	0.0523 (7)	
H9	0.5209	−0.0685	0.6403	0.063*	
C10	0.57666 (12)	−0.03518 (13)	0.4942 (3)	0.0616 (8)	
H10	0.5784	−0.0630	0.4704	0.074*	
C11	0.60844 (11)	0.00867 (13)	0.4287 (3)	0.0598 (8)	
H11	0.6317	0.0105	0.3617	0.072*	
C12	0.60606 (10)	0.04971 (12)	0.4617 (3)	0.0547 (7)	
H12	0.6279	0.0795	0.4183	0.066*	
C13	0.57083 (9)	0.04682 (11)	0.5604 (3)	0.0446 (6)	
C14	0.58649 (11)	0.12823 (12)	0.5356 (3)	0.0573 (7)	
H14	0.6062	0.1312	0.4551	0.069*	
C15	0.58292 (11)	0.16991 (11)	0.5738 (3)	0.0585 (7)	
C16	0.60823 (15)	0.21219 (14)	0.4862 (4)	0.0843 (11)	
H16	0.6253	0.2110	0.4038	0.101*	
C17	0.60896 (16)	0.25426 (14)	0.5161 (5)	0.0889 (11)	
H17	0.6255	0.2813	0.4543	0.107*	
C18	0.58475 (14)	0.25664 (13)	0.6400 (5)	0.0827 (11)	
H18	0.5858	0.2858	0.6635	0.099*	
C19	0.55925 (13)	0.21665 (13)	0.7291 (5)	0.0774 (10)	
H19	0.5428	0.2191	0.8112	0.093*	
C20	0.55721 (11)	0.17190 (11)	0.6999 (3)	0.0571 (7)	

### Atomic displacement parameters (Å^2^)

**Table:**

	*U*^11^	*U*^22^	*U*^33^	*U*^12^	*U*^13^	*U*^23^
Ni	0.0432 (2)	0.0485 (2)	0.0423 (2)	0.02653 (17)	0.00544 (14)	0.00408 (14)
O1	0.0591 (12)	0.0557 (12)	0.0528 (10)	0.0342 (10)	0.0125 (9)	0.0075 (9)
O2	0.0752 (15)	0.0579 (13)	0.0735 (13)	0.0390 (12)	0.0244 (11)	0.0102 (11)
O1W	0.104 (4)	0.077 (3)	0.073 (2)	0.047 (3)	0.0052 (19)	−0.0180 (17)
O1W'	0.085 (12)	0.046 (9)	0.123 (11)	0.041 (9)	0.002 (8)	−0.005 (7)
N1	0.0423 (12)	0.0541 (13)	0.0354 (10)	0.0255 (11)	−0.0041 (9)	−0.0004 (9)
N2	0.0407 (12)	0.0571 (14)	0.0417 (10)	0.0252 (11)	−0.0020 (9)	0.0009 (10)
C1	0.0427 (14)	0.0569 (16)	0.0363 (12)	0.0274 (13)	−0.0026 (11)	0.0060 (11)
C2	0.0486 (16)	0.0707 (19)	0.0419 (13)	0.0341 (15)	0.0008 (12)	0.0025 (13)
C3	0.0446 (15)	0.080 (2)	0.0409 (13)	0.0293 (16)	0.0013 (12)	0.0049 (14)
C4	0.0476 (16)	0.0648 (19)	0.0514 (15)	0.0172 (15)	0.0003 (13)	0.0110 (14)
C5	0.0515 (17)	0.0613 (18)	0.0531 (15)	0.0270 (15)	−0.0010 (13)	0.0019 (14)
C6	0.0429 (14)	0.0567 (16)	0.0391 (12)	0.0267 (13)	−0.0028 (11)	0.0027 (12)
C7	0.0473 (15)	0.0508 (15)	0.0419 (12)	0.0266 (13)	−0.0053 (12)	−0.0019 (12)
C8	0.0400 (14)	0.0608 (17)	0.0354 (12)	0.0284 (13)	−0.0068 (10)	−0.0063 (12)
C9	0.0538 (16)	0.0594 (18)	0.0474 (14)	0.0311 (15)	−0.0048 (13)	−0.0087 (13)
C10	0.066 (2)	0.076 (2)	0.0523 (16)	0.0428 (18)	−0.0050 (15)	−0.0175 (16)
C11	0.0526 (17)	0.084 (2)	0.0502 (15)	0.0399 (17)	0.0007 (13)	−0.0127 (15)
C12	0.0471 (16)	0.0686 (19)	0.0464 (14)	0.0274 (15)	0.0010 (12)	−0.0033 (14)
C13	0.0387 (14)	0.0627 (17)	0.0344 (11)	0.0269 (13)	−0.0063 (11)	−0.0053 (12)
C14	0.0477 (16)	0.066 (2)	0.0518 (15)	0.0237 (15)	0.0072 (13)	0.0060 (14)
C15	0.0519 (17)	0.0570 (18)	0.0622 (17)	0.0239 (15)	0.0021 (14)	0.0096 (14)
C16	0.088 (3)	0.072 (2)	0.085 (2)	0.034 (2)	0.020 (2)	0.021 (2)
C17	0.091 (3)	0.060 (2)	0.103 (3)	0.029 (2)	0.015 (2)	0.022 (2)
C18	0.079 (2)	0.054 (2)	0.115 (3)	0.0332 (19)	0.005 (2)	0.010 (2)
C19	0.068 (2)	0.059 (2)	0.108 (3)	0.0333 (18)	0.014 (2)	0.007 (2)
C20	0.0512 (17)	0.0544 (18)	0.0683 (18)	0.0283 (15)	0.0026 (14)	0.0082 (15)

### Geometric parameters (Å, °)

**Table:**

Ni—O1	1.8865 (18)	C6—C7	1.419 (4)
Ni—O2	1.886 (2)	C7—H7	0.9300
Ni—N1	1.930 (2)	C8—C9	1.379 (4)
Ni—N2	1.935 (2)	C8—C13	1.385 (4)
O1—C1	1.304 (3)	C9—C10	1.385 (4)
O2—C20	1.301 (3)	C9—H9	0.9300
O1W—O1W'	1.175 (15)	C10—C11	1.372 (5)
O1W—H1	0.829 (10)	C10—H10	0.9300
O1W—H2	0.841 (10)	C11—C12	1.367 (4)
O1W'—H2	0.39 (4)	C11—H11	0.9300
N1—C7	1.286 (3)	C12—C13	1.391 (4)
N1—C8	1.423 (3)	C12—H12	0.9300
N2—C14	1.289 (4)	C14—C15	1.417 (4)
N2—C13	1.411 (3)	C14—H14	0.9300
C1—C2	1.403 (4)	C15—C16	1.406 (5)
C1—C6	1.410 (4)	C15—C20	1.417 (4)
C2—C3	1.364 (4)	C16—C17	1.343 (5)
C2—H2A	0.9300	C16—H16	0.9300
C3—C4	1.384 (4)	C17—C18	1.378 (6)
C3—H3	0.9300	C17—H17	0.9300
C4—C5	1.362 (4)	C18—C19	1.368 (5)
C4—H4	0.9300	C18—H18	0.9300
C5—C6	1.407 (4)	C19—C20	1.406 (4)
C5—H5	0.9300	C19—H19	0.9300
			
O2—Ni—O1	87.61 (9)	C9—C8—C13	120.4 (2)
O2—Ni—N1	176.06 (9)	C9—C8—N1	124.8 (3)
O1—Ni—N1	94.63 (9)	C13—C8—N1	114.7 (2)
O2—Ni—N2	93.96 (9)	C8—C9—C10	118.9 (3)
O1—Ni—N2	176.40 (9)	C8—C9—H9	120.6
N1—Ni—N2	83.98 (9)	C10—C9—H9	120.6
C1—O1—Ni	127.01 (17)	C11—C10—C9	121.0 (3)
C20—O2—Ni	126.63 (19)	C11—C10—H10	119.5
O1W'—O1W—H1	122 (4)	C9—C10—H10	119.5
O1W'—O1W—H2	12 (4)	C12—C11—C10	120.2 (3)
H1—O1W—H2	110.1 (18)	C12—C11—H11	119.9
O1W—O1W'—H2	27 (8)	C10—C11—H11	119.9
C7—N1—C8	122.5 (2)	C11—C12—C13	119.9 (3)
C7—N1—Ni	124.63 (18)	C11—C12—H12	120.1
C8—N1—Ni	112.83 (17)	C13—C12—H12	120.1
C14—N2—C13	122.8 (2)	C8—C13—C12	119.6 (3)
C14—N2—Ni	124.3 (2)	C8—C13—N2	115.4 (2)
C13—N2—Ni	112.72 (17)	C12—C13—N2	124.9 (3)
O1—C1—C2	118.4 (3)	N2—C14—C15	125.8 (3)
O1—C1—C6	123.9 (2)	N2—C14—H14	117.1
C2—C1—C6	117.7 (3)	C15—C14—H14	117.1
C3—C2—C1	121.8 (3)	C16—C15—C20	118.4 (3)
C3—C2—H2A	119.1	C16—C15—C14	118.2 (3)
C1—C2—H2A	119.1	C20—C15—C14	123.3 (3)
C2—C3—C4	120.6 (3)	C17—C16—C15	123.1 (4)
C2—C3—H3	119.7	C17—C16—H16	118.5
C4—C3—H3	119.7	C15—C16—H16	118.5
C5—C4—C3	119.1 (3)	C16—C17—C18	118.7 (4)
C5—C4—H4	120.5	C16—C17—H17	120.6
C3—C4—H4	120.5	C18—C17—H17	120.6
C4—C5—C6	122.0 (3)	C19—C18—C17	120.9 (4)
C4—C5—H5	119.0	C19—C18—H18	119.5
C6—C5—H5	119.0	C17—C18—H18	119.5
C5—C6—C1	118.8 (2)	C18—C19—C20	121.8 (4)
C5—C6—C7	117.3 (3)	C18—C19—H19	119.1
C1—C6—C7	123.9 (3)	C20—C19—H19	119.1
N1—C7—C6	125.8 (3)	O2—C20—C19	118.9 (3)
N1—C7—H7	117.1	O2—C20—C15	124.1 (3)
C6—C7—H7	117.1	C19—C20—C15	117.1 (3)
			
O2—Ni—O1—C1	−178.2 (2)	Ni—N1—C8—C9	175.7 (2)
N1—Ni—O1—C1	−1.4 (2)	C7—N1—C8—C13	179.0 (2)
N2—Ni—O1—C1	65.8 (15)	Ni—N1—C8—C13	−3.1 (3)
O1—Ni—O2—C20	−162.7 (3)	C13—C8—C9—C10	0.4 (4)
N1—Ni—O2—C20	72.5 (13)	N1—C8—C9—C10	−178.3 (2)
N2—Ni—O2—C20	14.1 (3)	C8—C9—C10—C11	0.8 (4)
O2—Ni—N1—C7	123.7 (12)	C9—C10—C11—C12	−0.6 (4)
O1—Ni—N1—C7	−0.9 (2)	C10—C11—C12—C13	−0.9 (4)
N2—Ni—N1—C7	−177.6 (2)	C9—C8—C13—C12	−1.9 (4)
O2—Ni—N1—C8	−54.1 (13)	N1—C8—C13—C12	177.0 (2)
O1—Ni—N1—C8	−178.72 (16)	C9—C8—C13—N2	179.9 (2)
N2—Ni—N1—C8	4.61 (16)	N1—C8—C13—N2	−1.2 (3)
O2—Ni—N2—C14	−13.2 (2)	C11—C12—C13—C8	2.2 (4)
O1—Ni—N2—C14	102.7 (14)	C11—C12—C13—N2	−179.8 (2)
N1—Ni—N2—C14	170.2 (2)	C14—N2—C13—C8	−170.6 (2)
O2—Ni—N2—C13	171.37 (17)	Ni—N2—C13—C8	5.0 (3)
O1—Ni—N2—C13	−72.8 (14)	C14—N2—C13—C12	11.3 (4)
N1—Ni—N2—C13	−5.26 (16)	Ni—N2—C13—C12	−173.1 (2)
Ni—O1—C1—C2	−178.18 (17)	C13—N2—C14—C15	−178.6 (3)
Ni—O1—C1—C6	2.5 (4)	Ni—N2—C14—C15	6.4 (4)
O1—C1—C2—C3	−178.4 (2)	N2—C14—C15—C16	−178.5 (3)
C6—C1—C2—C3	1.0 (4)	N2—C14—C15—C20	4.8 (5)
C1—C2—C3—C4	0.3 (4)	C20—C15—C16—C17	−0.4 (6)
C2—C3—C4—C5	−1.0 (4)	C14—C15—C16—C17	−177.2 (4)
C3—C4—C5—C6	0.5 (4)	C15—C16—C17—C18	1.5 (6)
C4—C5—C6—C1	0.8 (4)	C16—C17—C18—C19	−1.8 (7)
C4—C5—C6—C7	179.8 (3)	C17—C18—C19—C20	1.0 (6)
O1—C1—C6—C5	177.8 (2)	Ni—O2—C20—C19	171.7 (2)
C2—C1—C6—C5	−1.5 (4)	Ni—O2—C20—C15	−7.9 (4)
O1—C1—C6—C7	−1.1 (4)	C18—C19—C20—O2	−179.4 (3)
C2—C1—C6—C7	179.5 (2)	C18—C19—C20—C15	0.2 (5)
C8—N1—C7—C6	179.9 (2)	C16—C15—C20—O2	179.1 (3)
Ni—N1—C7—C6	2.3 (4)	C14—C15—C20—O2	−4.2 (5)
C5—C6—C7—N1	179.6 (2)	C16—C15—C20—C19	−0.5 (5)
C1—C6—C7—N1	−1.5 (4)	C14—C15—C20—C19	176.2 (3)
C7—N1—C8—C9	−2.1 (4)		

### Hydrogen-bond geometry (Å, °)

**Table:**

*D*—H···*A*	*D*—H	H···*A*	*D*···*A*	*D*—H···*A*
O1w—H1···O1	0.83 (1)	2.06 (2)	2.842 (4)	158 (5)
